# Evaluation of the French National Program on Home Return of Patients with Chronic Heart Failure (PRADO-IC): Pilot Study of 91 Patients During Its Deployment in the Bas Rhin Area

**DOI:** 10.3390/jcm9041222

**Published:** 2020-04-23

**Authors:** Mylène Radreau, Noel Lorenzo-Villalba, Samy Talha, Jean-Jacques Von Hunolstein, Michel Hanssen, Anne Koenig, Philippe Couppie, Bernard Geny, Francois Severac, Gérald Roul, Abrar-Ahmad Zulfiqar, Emmanuel Andrès

**Affiliations:** 1 Service de Médecine Interne, Diabète et Maladies Métaboliques, Clinique Médicale B, Hôpitaux Universitaires de Strasbourg, 67000 Strasbourg, France; mylene.radreau@chru-strasbourg.fr (M.R.); noellorenzo@gmail.com (N.L.-V.); emmanuel.andres@chru-strasbourg.fr (E.A.); 2 Service de Physiologie et Laboratoire d’Explorations Fonctionnelles, Hôpitaux Universitaires de Strasbourg, 67000 Strasbourg, France; samy.talha@chru-strasbourg.fr (S.T.); bernard.geny@chru-strasbourg.fr (B.G.); 3 Equipe de recherche EA 3072 “Mitochondrie, Stress oxydant et Protection musculaire”, Faculté de Médecine de Strasbourg, Université de Strasbourg, 67000 Strasbourg, France; 4 Service de Cardiologie, Unité Fonctionnelle dédiée à l’insuffisance cardiaque, Pôle Médico-chirurgical de Cardiovasculaire, Hôpitaux Universitaires de Strasbourg, 67000 Strasbourg, France; jean-jacques.vonhunolstein@chru-strasbourg.fr (J.-J.V.H.); anne.koenig@chru-strasbourg.fr (A.K.); gerald.roul@chru-strasbourg.fr (G.R.); 5 Service de Cardiologie, Centre Hospitalier de Haguenau, 67500 Haguenau, France; michel.hanssen@ch-haguenau.fr (M.H.); philippe.couppie@ch-haguenau.fr (P.C.); 6 Département de santé publique et d’épidémiologie, Hôpitaux Universitaires de Strasbourg, 67000 Strasbourg, France; francois.severac@chru-strasbourg.fr

**Keywords:** heart failure, return home program, hospitalization, mortality, care pathway, treatment

## Abstract

Objective: The main objective of this study was to evaluate the impact of the French national program on home return of chronic heart failure patients (PRADO-IC) in terms of re-hospitalizations for heart failure (HF) during its deployment in the Bas-Rhin (France). Patients and methods: This was a pilot, descriptive, quantitative, retrospective, and bi-centric study (University Hospitals of Strasbourg and Haguenau Hospital Center, France). It included all patients included in the PRADO-IC program from these centers between January 1, 2015 and December 31, 2015. The primary endpoint of our study was the evaluation of the number of 1-year, 6-month, and 30-day re-admissions to the hospital in relation to an acute HF episode, before and after the inclusion of patients in the PRADO-IC program. The secondary endpoints were the number of overall re-hospitalizations (all-cause); the number of days of hospitalization for HF; the time to first re-hospitalization and the average length of hospital stay, before and after inclusion in PRADO-IC; and the overall and cardiovascular mortality rates. Results: 91 patients out of 271 (33,6%) with a mean age of 79.2 years (67–94) were included. They all had chronic HF, essentially class II-III NYHA (90.1%), mostly of ischemic origin (41.9%), with altered left ventricular ejection fraction in 71.4% of cases. A reduction in the mean number of hospitalizations for HF per patient at 30 days, 6 months and 1 year was observed, respectively, from 0.18 ± 0.42 per patient before inclusion to 0.15 ± 0.36 after inclusion (*p* = 0.56); 0.98 ± 1.04 hospitalizations to 0.53 ± 0.81 at 6 months (*p* < 0.01); and 1.64 ± 1.14 hospitalizations 1.04 ± 1.05 at 1 year (*p* < 0.001). Patients were hospitalized less overall after inclusion in the PRADO-IC program. The number of days of hospitalization for HF was reduced after inclusion of patients from 18.02 ± 7.78 days before inclusion to 14.28 ± 11.57 days for the 6 month follow-up (*p* = 0.006), and from 22.07 ± 10.33 days before inclusion to 16.39 ± 15.94 days for the 1 year follow-up (*p* < 0.001). In contrast, inclusion in PRADO-IC statistically increased the mean time to first re-hospitalization for HF from mean 99.36 ± 72.39 days before inclusion to 148.11 ± 112.77 days after inclusion (*p* < 0.001). Conclusion: This study seems to demonstrate that the PRADO-IC program could improve the management of chronic HF patients in ambulatory care, particularly regarding HF re-hospitalization. However, due to the limitations of the methodology used and the small number of patients, it is advisable to consolidate its initial results with a randomized controlled study on a larger number of patients. In our opinion, its results need to be communicated because, to our knowledge, no equivalent study exists.

## 1. Introduction

The prevalence of heart failure (HF) in France is estimated at 2.3% of the population, which corresponds to approximately 1,130,000 people [1]. This number of people has increased by 30% in 10 years. Each year, 120,000 to 150,000 new cases are diagnosed [2]. The prevalence of HF increases sharply with age, especially after the age of 65 [3].

HF is a chronic disease, characterized by episodes of acute decompensation leading to recurrent hospitalizations, and is responsible for a high mortality rate [1,3]. In this context, 25% of HF patients are readmitted within 30 days after a hospitalization for acute HF and 50% during the year. One third of patients with HF die within 1 year of first hospitalization for cardiac decompensation and 40% within 2 years [3].

In France, HF represents one of the main causes of hospitalization in individuals aged 65 or more [4]. The annual cost of hospitalizations due to HF is estimated at more than one billion euros, representing 63% of the healthcare costs reimbursed for patients with long-term illness (LTID) [5]. Because of the aging population, the prevalence and costs of HF in France are constantly increasing, subsequently representing a major public health issue.

In 2004, the Public Health Law included in its objectives the reduction of cardiac decompensation rates [6]. Several studies and clinical surveys suggest that a certain number of hospitalizations could be avoided thanks to multidisciplinary intervention programs implemented during hospitalization, at the time of discharge, or during outpatient follow-up. [7,8,9].

To this end, the National Health Insurance Fund (*Caisse Nationale d’Assurance Maladie* [CNAM]) in 2013 set up a home return program for patients hospitalized for HF decompensation (*Programme de Retour A Domicile des patients Insuffisants Cardiaques* [PRADO-IC]) in order to improve the post-hospital transition and coordination between private health care professionals [10]. This program was deployed in the Bas-Rhin Department (France) in 2014.

The objective of this study was to evaluate the PRADO-IC program with respect to its effectiveness in HF, in particular on re-admission rates in patients with HF, based on a pilot study.

## 2. Patients and Method

### 2.1. Objective

The main objective was to assess the impact of the PRADO-IC program [10], administered by the CNAM, on re-hospitalizations for HF during the deployment of the program in the Bas-Rhin (France). The secondary objective was to describe the characteristics of the Bas-Rhin population participating in this program.

### 2.2. The PRADO IC Program

The PRADO-IC program is a national home return program for patients hospitalized for HF decompensation aiming at improving the post-hospital transition and coordination between private health care professionals [10]. This program was deployed in the Bas-Rhin Department (France) in 2014, as this region was selected as a pilot for optimizing the management of CHF patients. The PRADO-IC approach aims to reduce the rates of re-hospitalization (30%) and mortality (20%) [10]. To achieve this, it is based on three main axes:

(1) Initialization of medical monitoring, in order to respond to the patient’s request by allowing him or her to return home as soon as hospitalization is no longer necessary;
(2) Supporting the patient and strengthening post-hospitalization follow-up;

Optimization of home return, with the implementation of assistance if necessary.

(3) The duration of PRADO-IC support varies according to severity as assessed by the New York Heart Association (NYHA) classification [10]. A patient in NYHA class I or II receives home support for 2 months. Patients in NYHA class III and IV HF receive home support for a maximum duration of 6 months (renewable for 2-month periods).

The PRADO-IC program includes:

(1) A consultation with the attending physician within 8 days after returning home and another consultation within 2 months;
(2) A weekly monitoring and education visit by the nurse during 2 months for all patients, and a visit every 2 weeks for 4 months for patients in class III and IV;
(3) A consultation during the 2nd month with the cardiologist [10].

The visiting nurses involved in this program receive prior training carried out in the form of e-learning by the French Cardiology Society (*Société Française de Cardiologie* [SFC]) [10]. The general practitioner receives benchmarks of good practice on the course of care, the titration of drugs, their monitoring, the management of their side effects and the action to be taken in case of worsening.

In addition, a follow-up notebook is given to each patient included in the program, useful as a support for education and self-monitoring for the patient and as an element of coordination between healthcare professionals [10].

### 2.3. Type of Study

This was a pilot, descriptive, quantitative, retrospective, and bicentric study, with the participation of a University Hospital and a General Hospital. The supervisors in charge of the PRADO-IC program determined the design of the study, mainly due to time and financial constraints. Ideally, a randomized controlled study had been considered, or a study versus a control group, but this was not possible because medical data from the CPAM (*Caisse Primaire d’Assurance Maladie*) was no longer available for patients not included in PRADO-IC. For this reason, a study comparing the clinical behavior of patients before and after the inclusion in PRADO-IC program was launched. This was considered more appropriate than comparing the population studied with data extrapolated from the French population. The medical teams involved included:

(1) The internal medicine, diabetes and metabolic diseases service of Medical Clinic B at Strasbourg University Hospitals (*Hôpitaux Universitaires de Strasbourg* [(HUS], Strasbourg, France);
(2) The cardiology service of the New Civil Hospital through the functional unit dedicated to HF patients at HUS;
(3) The cardiology service of Haguenau Hospital Center (*Centre Hospitalier de Haguenau* [CHH], Haguenau, France).

### 2.4. Study Population and Inclusion Criteria

The patients included in this study were all patients who participated in the PRADO-IC program between January 1 and December 31, 2015, after hospitalization in one of the two previously mentioned medical facilities.

The patients eligible for the PRADO-IC program were chronic HF patients, hospitalized for an episode of HF decompensation, including patients of both sexes, aged over 18 years, and who agreed to participate [10]. Diagnosis of chronic HF was established by the clinicians in charge of the patients based on the clinical, biological, and ultrasound criteria of the European Society of Cardiology (ESC) [11]. Hospital discharge and subsequent enrollment in the program was considered taking into account different parameters: absence of dyspnea within 48 hours prior to discharge, absence of crackles, regression of edemas, return to baseline or ideal weight (the latter being determined by the patient’s usual cardiologist), return to blood pressure and heart rate, precipitating factor (if identified) controlled, comorbid conditions evaluated, renal function and electrolytes steady, ventricular ejection fraction known, and treatment adapted. Inclusion criteria also include a patient’s ability to perform daily life activities.

Lack of agreement from the patient’s primary doctor or cardiologist was main exclusion criterion. The other exclusion criteria for the PRADO-IC program included medical criteria and autonomy criteria, as well as the patient’s refusal to participate [10]. The medical criteria for ineligibility were kidney dialysis; short-term surgical treatment; significant cognitive dysfunction; pending heart transplant; need for transfer to a care and rehabilitation service (CRS); and need for palliative care.

The autonomy criteria for non-eligibility were an inability to get up, lie down, or sit alone; inability to understand instructions (behavioral disorder); an inability to walk alone in one’s home (need for a third person); and a need for transfer to a specialized institution (accommodation establishment for dependent elderly person). The patient had to be affiliated with the general health insurance scheme.

**Figure 1:**
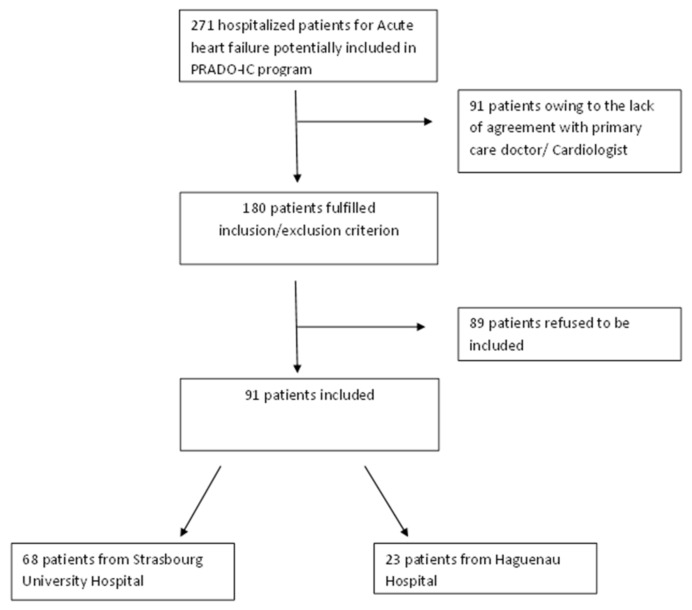
Patient’s flow chart.

Two hundred and seventy-one patients were initially identified to join the PRADO-IC program (prescreen) because they had health insurance coverage (CPAM) and had given their consent (Figure 1). All these patients were hospitalized at the University Hospital of Strasbourg and at the Haguenau Hospital. Ninety-one patients (33.6%) were excluded from the study due to the refusal of either the general practitioner or the city cardiologist to participate in the PRADO-IC program. In addition, 89 patients (32.8%) were not included because they did not meet the autonomy criteria for home return and/or were frail. Finally, only 91 patients (33.6%) were included in the PRADO-IC program.

### 2.5. Outcome Criteria

#### 2.5.1. Main Outcome Criterion

The main endpoint of our study was the evaluation of the number of readmissions for HF at 1 year, 6 months, and 30 days, before and after inclusion in the PRADO-IC program [10].

#### 2.5.2. Secondary Outcome Criteria

The number of overall re-hospitalizations (all causes combined) and other causes, the number of days of hospitalization for HF, delay before the first re-hospitalization, and the average length of hospital stay (ALHS) before and after inclusion in the PRADO-IC program [10];

Total and cardiovascular mortality;

The evaluation of the optimization of cardiovascular therapies, comparing the treatment at inclusion and at 6 months;

An estimation of the overall cost, including expenditures on nursing care, medical consultations, and pharmaceutical costs.

### 2.6. Data Collection

This study was a retrospective review of electronic medical records of the patients included in the PRADO-IC program [10] between January 1 and December 31, 2015. The data were collected by a single investigator over a period of 6 months from January 1, 2017 and were anonymously stored on a computer host approved for medical data. This collection was carried out using Dx-care software from Medasys (Paris, France) within the HUS and the CHH.

For each patient, the following data were collected: age, sex, date of death and cause, date of inclusion in PRADO-IC, department that carried out the inclusion, cardiovascular risk factors, co-morbidities, HF etiology, medical treatment and doses, left ventricular ejection fraction (LVEF), NYHA class, biological characteristics at inclusion, and number and type of hospitalizations before and after the date of inclusion in the PRADO-IC program. For each hospitalization, we collected the dates of admission and discharge, reason, medical treatments, and doses of medications (at admission and discharge).

Events occurring after December 31, 2016 were not collected, nor were those occurring before January 1, 2014. The missing data were supplemented by a telephone call to the attending physician.

The reasons for hospitalizations were categorized as “causes of heart failure”, that is, those related to cardiac decompensation, or “all causes”. The same categories were used for mortalities.

A cost estimation was made by collecting and cross-referencing the data from the CNAM database with that of the Medical Service for Health Insurance (*Service Médical de l’Assurance Maladie*), with regard to the targeted economic indicators.

### 2.7. Administrative Documents

Approval for the study was obtained from the Ethics Committee of the Faculty of Medicine of Strasbourg (Strasbourg, France) after an application dated on March 18, 2016 (FC / file 2016-67), and a declaration to the National Commission for Information Technology and Liberties (*Commission Nationale Informatique et Liberté* [CNIL]) was carried out on April 6, 2016 (N ° 1945926 v 0). The study was referenced in ClinicalTrials.gov (ID: NCT0280822).

### 2.8. Statistical Analysis

The statistical method was descriptive. Quantitative variables were expressed as means and standard deviations; the extremes were indicated. The Wilcoxon test for paired series was used to compare values within the same sample before and after inclusion in the statistical analysis. Each case was its own control, and, to facilitate comparability, a death match was made.

For all statistical analyses, the significance threshold was set at 0.05.

## 3. Results

Between January 1 and December 31, 2015, 91 patients out of 271 (33,6%) were included in the present study and subsequently in the PRADO-IC program after hospitalization for acute HF. Sixty-eight patients (74.7%) were recruited from the HUS and 23 patients from the CHH.

### 3.1. Characteristics of the Population

#### 3.1.1. Main Features

The study population consisted of 51 males and 40 females with a mean age at inclusion of 79.2 ± 10.2 years (67–94 years).

Most patients had one or more comorbidities, and most had several cardiovascular risk factors: high blood pressure (hypertension) in 92.3% of cases (n = 84), dyslipidemia in 79.1% (n = 72), chronic renal failure (defined by a creatinine clearance <60 ml/min according to CKD-EPI) in 61.5% (n = 56), coronary disease in 47.3% (n =43), diabetes in 45.1% (n = 41), chronic obstructive pulmonary disease (COPD) in 34.1% (n = 31), proven vascular disease (stroke, obliterating arterial disease of the lower limbs) in 25.3% (n = 23), and cancer progressing over the past 5 years in 15.4% of cases (n = 14). Table 1 presents the general characteristics of the studied population.

Of note, the mean BMI was 27.4 ± 2.3 kg/m2, and 25.3% (n = 23) were classified as obese (defined by a BMI > 30 kg/m2).

Atrial fibrillation was present at admission in 62.6% of patients (n = 57). Nineteen patients (20.9%) had a pacemaker and 12 (13.2%) had an automatic defibrillator.

#### 3.1.2. Data Concerning Heart Failure

The main etiology of the HF was ischemic heart disease in 41 patients (45.1%); heart disease secondary to hypertension in 13 subjects (14.3%); and valvular heart disease in ten individuals (11%) (Table 1). Twelve patients (13.2%) were found to have any kind of cardiac arrhythmia (without reliable documentation). The other types of heart disease were toxic (8.8%, n = 8), primary (2.2%, n = 2), and of unknown etiology (5.5%, n = 5).

The majority of patients were in NYHA class II (52.7%, n = 48); 37.4% (n = 34) were in class III and 9.9% (n = 9) in class IV (Table 1). The LVEF was preserved (≥50%) in 28.6% of the subjects (n = 26) and significantly abnormal (<30%) in 38.5% of the patients (n=35). A LVEF between 35% and 50% was noted in 32.9% of the cases (n = 30).

Upon admission in the study, mean BNP or NT pro-BNP were 968.5 ± 743.7 pg/mL (158–1487) and 13218.4 ± 8139.5 pg/mL, respectively (2056–27829). After hospital discharge and inclusion in PRADO-IC program, the mean BNP and NT pro-BNP were 468.8 ± 968.5 pg/mL (123–1205) and 7053.6 ± 706.2 pg/mL (2.102–1489), respectively.

Among the triggers of cardiac decompensation, infection was the main cause of cardiac decompensation at baseline in 23.1% of patients (n = 21), followed by arrhythmia (especially atrial fibrillation) in 18.7% of subjects (n = 17), acute myocardial ischemia (9.9%, n = 9), and an inappropriate sodium diet (9.9%, n = 9) (Table 1). Acute renal failure (defined by creatinine clearance < 30 ml/min according to CKD-EPI) and uncontrolled hypertension were each found in eight (8.8%) patients. Non-compliance with therapy was observed in 7.7% (n = 7). Acute anemia was seen in 4.4% of cases (n = 4) while 54 patients (59.3%) had chronic anemia. Finally, 8.8% of patients (n = 8) did not have any identified triggers.

#### 3.1.3. Data Concerning Heart Failure Treatment

The PRADO-IC program included the prescription of β-blocker (bisoprolol, metoprolol, nebivolol, carvedilol) in 84.6% of patients (n = 77), angiotensin-converting enzyme inhibitor (ACE, mainly ramipril) in 57.1% (n = 52) of cases, and angiotensin II receptor antagonist (ARA II, mainly valsartan) in 23.1% of cases (n = 21) (Table 2). Most patients (90.1%, n = 82) were taking a loop diuretic and 41.8% (n = 38) were receiving anti-aldosterone therapy (spironolactone, epleronone). A combination of converting enzyme inhibitor or ARA II with a β-blocker was observed in 69.2% of patients (n = 63), and treatment with anti-aldosterone was added in 32.9% of those patients (n = 30). Twenty-five patients (27.5%) were on amiodarone, 14 (15.4%) were on a dihydropyridine drug, 11 (12.1%) were on digitalis, nine were on nitrates (9.9%), and seven patients (8.7%) were taking ivabradine. Fifty-six patients (61.5%) were receiving antiplatelet therapy and 55 (60.4%) were receiving anticoagulant therapy.

### 3.2. Outcomes Criteria

#### 3.2.1. Main Outcome Criterion

The mean number of hospitalizations for HF globally decreased and was statistically significant at six months and one year before and after inclusion in the program: 0.98 ± 1.04 hospitalizations versus 0.53 ± 0.81 at six months (*p* < 0.01) and 1.64 ± 1.14 hospitalizations versus 1.04 ± 1.05 at one year (*p* < 0.001). At 30 days, a reduction of hospitalizations per patient was also observed (0.18 ± 0.42 versus 0.15 ± 0.36, *p* = 0.56) (Table 3).

#### 3.2.2. Secondary Outcomes Criteria

The average number of readmissions other than HF decompensation per patient was significantly reduced at 30 days, 6 months, and 1 year after inclusion: 0.16 ± (−0.37) hospitalizations versus 0.02 ± 0.15 at 30 days (*p* < 0.001); 0.52 ± 0.72 hospitalizations versus 0.28 ± 0.59 at 6 months (*p* = 0.006) and 0.92 ± 1.04 hospitalizations versus 0.48 ± 0.78 at 1 year (*p* = 0.002) (Table 4).

The length of hospitalization for HF was also reduced after patients were included in the PRADO-IC program; 11.67 ± 4.34 days before inclusion versus 9.77 ± 3.92 days after inclusion at 30 days (*p* = 0.31). As before, this reduction became statistically significant at 6 months and 1 year (Table 4). Thus, the number of days spent in hospital for HF was 18.02 ± 7.78 days before inclusion versus 14.28 ± 11.57 days after inclusion for follow-up at 6 months (*p* = 0.006) and 22.07 ± 10.33 days before inclusion versus 16.39 ± 15.94 days after inclusion for 1 year follow-up (*p* < 0.001).

Inclusion in PRADO-IC program statistically lengthened the average delay before the first re-hospitalization for HF: an average delay of 99.36 ± 72.39 days before inclusion versus a delay of 148.11 ± 112.77 days after inclusion (*p* < 0.001). Of the 91 patients, 25 patients (27.5%) died within 1 year of inclusion. Thirteen deaths were related to HF (14.3%), including one within 30 days of discharge, five in the first 6 months, and seven in the following 6 months.

Evaluation of pharmacotherapy revealed non-statistically-significant changes in the main HF drugs at 6 months in the percentage of prescriptions of drug classes: 84.6% of patients on β-blocker at the end of hospital versus 76.2% at 6 months; 57.1% of patients on IEC versus 46.2%; 23.1% on ARB II versus 21.2%; and 41.8% of patients on anti-aldosterone versus 41.2% (all *p* not significant).

However, an optimization of the different classes was observed in almost 20% to 30% of the cases, with an optimal dose reached in 5% to 10% of the cases according to the different therapeutic classes considered. The main causes of non-optimization were acute renal failure or worsening of chronic renal failure in 13 patients (14.3%), orthostatic hypotension in 12 (13.2%), worsening of the HF in six (6.6%), hyperkalemia in five (5.5%), and bradycardia in four patients (4.4%).

Upon evaluation of death rates, 42 patients benefited from a 2-month follow-up in the PRADO-IC program and 34 from a 6-month follow-up. In this context, nursing health expenditure was estimated at €161,300 and pharmaceutical expenditure at €86,181. Regarding medical follow-up, 47% of patients had an evaluation by the cardiologist at least once in the 6 months following hospitalization and 97% consulted a general practitioner.

## 4. Discussion

This study showed that deployment of the PRADO-IC program [10] is possible among patients hospitalized for acute cardiac decompensation. This study, despite a criticizable methodology used (retrospective analysis before and after the implementation of the PRADO-IC program), seems to show favorable results on the management and outcome of heart failure patients. It seems therefore important to consolidate these results through a randomized controlled study. These results need to be communicated because, to our knowledge, no equivalent study exists. This study showed a reduction in the number of HF hospitalizations per patient at 6 months (a reduction of 5.9%) and 1 year (a reduction of 36.6%). On average, a reduction of about 33% in the number of re-hospitalizations for HF was found, which met one of the objectives of the program [10,12].

The results obtained in this study with the PRADO-IC program are particularly interesting in view of the high rate of hospitalization and re-hospitalization for HF reported in the literature, especially for patients in NYHA classes II-III [4,13,14]. Almost 50% of the patients in this cohort were in NYHA classes III and IV and were at very high risk for re-hospitalization [4,5]. According to the literature, after initial hospitalization for HF decompensation, the risk of readmission rises from 40% to 60% during the first year for NYHA class III and IV [4,13,14]. A previous French study showed that, out of 152,601 patients followed up after a first hospitalization for HF decompensation during 2009, 25% of patients were readmitted within 30 days and 50% in the year [3].

The population studied, although highly selected by the protocol of the PRADO-IC program (33.6% of the initial selected population), reflects the French population of patients with chronic HF in terms of demographic and medical characteristics [1,3,13]. The mean age of our patients was 79.3 years, and more than a third were over 85 years old. The majority of this group were males. Our cohort is made up of fragile and multi-pathological patients, with many cardiovascular risk factors. Coronary artery disease represents the main cause of HF in the patients studied (45.1%), followed by hypertension (14.3%), consistent with current epidemiological data [1,2,13].

The treatment of our patients at inclusion generally followed the recommendations of the ESC [11], since the majority of them had optimal drug treatment combining an ACE inhibitor or ARA2 with a β-blocker and an anti-aldosterone agent. It should be noted that newer pharmacological agents that have shown efficacy in HF (sacubitril/valsartan, empagliflozin) [15] were not available at the time of the study in 2015. The data seem to be consistent with those reported in the national ODIN study (*observatoire de l’insuffisance cardiaque* study) [16].

In our study, the triggers for acute HF were infections (23.1%), followed by rhythm disturbances (18.7%) and acute myocardial ischemia (9.9%). The French EFICA study found myocardial ischemia first, followed by arrhythmias and infections [17]. The triggers most frequently listed by the French National Authority for Health (*Haute Autorité de Santé* [HAS]) are poor adherence to treatment or excessive water and sodium intake, followed by heart rhythm disorders, ischemic heart disease, and finally inappropriate control of cardiovascular risk factors such as hypertension [18].

In the present study, HF decompensation was attributed to an inappropriate regime in 9.9% of the cases and to therapeutic non-compliance in 5.5%. However, assessing therapeutic adherence is difficult, especially in the elderly, where risk factors for non-compliance multiply (high number of drugs, cognitive disorders, isolation or poor socio-economic conditions, non-adherence to treatment, or an increase in the number of side effects) [19,20]. In addition, these low rates are probably explained by the exclusion of patients with cognitive impairment from the PRADO-IC program. In this context, an early multidisciplinary and educational approach has shown its effectiveness in improving compliance and reducing re-hospitalizations in these patients [21,22].

The favorable results observed regarding HF in our study might have several explanations related to the PRADO-IC program:

(1) Anticipation and organization of patients returning home after hospitalization;
(2) Establishment of a care pathway based on urban health professionals and their awareness;
(3) At-least-weekly monitoring of patients, allowing early detection of the warning signs of heart decompensation;
(4) Better adherence to dietary measures and treatments as well as their optimization and the absence of therapeutic inertia [18,23].

We have documented an optimization of drug treatment in 20% to 30% of patients. Other studies have shown the effectiveness of this type of program, using outpatient intervention conducted by the attending physician and home monitoring by specialized nurses [7,8,24,25].

A recent Cochrane review by Takeda et al., showed that intensive follow-up by specialized nurses at home significantly reduces readmissions for HF at one year [7]. Another meta-analysis confirmed these results, showing a reduction of HF readmissions at 3 and 6 months resulting from home nurse intervention [8]. In addition, early post-hospital medical follow-up, starting less than 7 days after discharge, along with a high frequency of contact between patients and caregivers, helps to reduce the readmission rate [24,25].

In France, various projects and tools have been developed to optimize the management of chronic HF. Health professionals from different health care facilities have created ambulatory follow-up programs for HF patients known as health networks (for example: the Heart Failure Network [RESICARD] in the Haut de Seine region and the Heart Failure Network in Lorraine [ICALOR]) [26,27,28,29,30]. The common objective of these networks is to improve the coordination of care for HF patients by facilitating communication between caregivers. Their goal is also to provide therapeutic education. In this context, the ICALOR network reported particularly interesting results, with a reduction of 7.19% in hospitalizations for HF compared to the number of hospitalizations expected over the same period (n = 1222; 48% and 32% NYHA class II and III patients, respectively) [29].

In this context, telemedicine is also a promising tool in the optimization of follow-up in HF patients (for a complete review of the literature, see [31]). Recently, the Telemedical Interventional Management in Patients with Heart Failure (TIM-HF2) study demonstrated, in a prospective randomized study, the usefulness of telemedicine (n = 1571) [32]. In the TIM-HF2 study, the all-cause mortality rate was 7.86 [95% CI: 6.14–the standard care group (RR = 0.70 [95% CI: 0.50–0.96]; *p* = 0.0280).

The percentage of all-cause deaths in our population was 3.3% at 30 days after inclusion, 12.1% at 6 months, and 27.5% at 1 year. Mortality rates attributed and not attributed to HF were almost similar. These results seem be in line with the patients’ phenotype and consistent with current epidemiological data [17,33,34]. In 2009, a French national study analyzed patient outcomes two years after a first hospitalization for HF. A total of 69,958 patients were enrolled, mean age 78 years. The death rate (from all causes) was 11% at one month, 29% at one year and 40% at two years [33]. In a recent report by the *Institut de Veille Sanitaire* (InVS), the mortality rate in France in HF patients dropped by around 36% between 2000 and 2013 [13]. In males over 65 years, it dropped from 952 deaths per 100,000 to 618 and in females over 65 years, from 644 deaths per 100,000 to 409. Due to the study design, we were unable to study whether inclusion in the PRADO-IC program would reduce this mortality rate.

In relation to the medical and pharmaceutical consumption in our population, the overall cost of nursing expenses in ambulatory care was estimated at €161,300 (average €1,967 per patient) and pharmaceutical expenses at €86,181 (average €968 per patient) for a 6-month period after their inclusion. The average annual reimbursement for long-term illness patients with HF was estimated at €921 per patient for nursing procedures, and €1,392 for pharmacy in 2007 in France [18]. We observed an increase in the consumption of nursing and medicines in our sample. This seems consistent with the objectives of PRADO-IC aimed at strengthening home monitoring and treatment optimization.

These costs should be compared with the savings made in terms of hospitalization and re-hospitalization for HF, the latter being reduced by 45.9% at 6 months (average cost of hospitalization for HF €4,500). In 2016, the CNAM spent 0.9% of its expenditure on acute HF [5]: 1,447 million euros, of which 77% were devoted to hospital expenditure—1,097 million euros over one year.

A retrospective study carried out by Jourdain et al., comparing the follow-up of chronic HF patients after hospitalization for acute episodes in therapeutic HF Units versus the usual care at home, showed therapeutic HF Units significantly reduce the risk of re-hospitalization, by approximately 28%, but also significantly reduce, by more than 50%, the costs linked to hospitalizations, regardless of the co-morbidities or the severity of the pathology [35]. In addition, the intervention of the Lorraine network ICALOR, cited above, has enabled an estimated reduction of around two million euros in hospital costs, secondary to the decrease in the number of HF readmissions [29].

Our study has some limitations. First, it is a pilot, retrospective, observational study, where each case is its own control without a control group, resulting in a low level of evidence and an increased risk of missing data. In addition, only hospitalizations from Strasbourg University Hospital or Haguenau Hospital were taken into consideration, as they are expert and referral centers. Our favorable results on re-hospitalizations might therefore be overestimated.

The before/after design also does not allow us to be certain about the reasons for readmission decreases or the average length of stay, which might also be linked to other factors such as the evolution of treatments or other associated comorbidities. Nevertheless, all patients had identical follow-up durations (30 days, 6 months, 1 year) before and after inclusion in PRADO-IC, and we matched them based on the deaths in order to reduce bias. Interpretation of our data regarding the impact on death rates is difficult due to the presence of several factors leading to death.

Even if our sample characteristics are generally in line with current epidemiological data, we should be careful when extrapolating these results. The small population size (91 patients) was due to low recruitment by the hospital teams in 2015, as the PRADO-IC program started in February 2013. In addition, there were many exclusion criteria in the study. Elderly people with cognitive impairment, in dialysis, or with a loss of autonomy were excluded from the PRADO-IC program, making our sample difficult to compare with the general population. This study does not allow us to suggest the duration of follow-up in HF patients, even though we would be tempted to say that it should be maintained in the long term (proposed between the second and sixth month or reduced depending on the results obtained).

## 5. Conclusions

This study seems to demonstrate that the PRADO-IC program might improve the management of chronic HF patients in ambulatory care, particularly regarding HF re-hospitalization. A reduction in the number and delay of re-hospitalizations for HF in the patients enrolled was observed. Besides, its impact on treatment optimization was moderate but present, resulting in an increase in the number of patients treated with an optimal dosage but with an unchanged prescription rate. However, due to the limitations of the methodology used and the small number of patients, it is advisable to consolidate its initial results through a randomized controlled study on a larger number of patients. 

## 6. Recommendations

These results encourage the continuation of the development of PRADO-IC. Additional evaluations, in particular using prospective monitoring or integrating a quality-of-life questionnaire, would be helpful to adjust the follow up modalities.

## Figures and Tables

**Table 1:** General characteristics of the population studied.

Variable	n = 91	%
**Medical antecedents**		
High blood pressure	84	92.3
Dyslipidemia	72	79.1
Atrial Fibrillation	57	62.6
Chronic renal failure	56	61.5
Coronary disease	43	47.3
Diabetes	41	45.1
COPD	31	34.1
Obesity	23	25.3
Proven vascular disease	23	25.3
Pacemaker	19	20.9
Cancer in progression (>5 years)	14	15.4
Automatic defibrillator	12	13.2
**NYHA Classification**		
NYHA class II	48	52.7
NYHA class III	34	37.4
NYHA class IV	9	9.9
**Heart failure etiology**		
Ischemic heart disease	41	45.1
Hypertension	13	14.3
Valvular heart disease	10	11
Arrhythmia	12	13.2
Toxic	8	8.8
Primary	2	2.2
Unknown etiology	5	5.5
**LVEF**		
<30%	35	38.5
35–50%	30	32.9
>50%	26	27.5
**Precipitating factor**		
Chronic anemia	54	59.3
Infection	21	23.1
Arrhythmia	17	18.7
Acute myocardial ischemia	9	9.9
Inappropriate sodium diet	9	9.9
Acute renal failure	8	8.8
Uncontrolled hypertension	8	8.8
Non-compliance with therapy	7	7.7
Acute anemia	4	4.4
No identified	8	8.8

COPD: Chronic obstructive pulmonary disease, NYHA: New York Heart Association Functional Classification, LVEF: left ventricular ejection fraction.

**Table 2:** Medical treatment upon enrollment in the PRADO-IC program.

Treatment	n = 91%	
**Loop diuretic**	82	90.1
**β-blocker**	77	84.6
**ACEI**	52	57.1
**ARABs**	21	23.1
**Aldosterone Antagonists**	38	41.8
**ARABsI+ β-blocker**	63	69.2
**ARABs+ β-blocker+ Aldosterone Antagonists**	30	32.9
**Amiodarone**	25	27.5
**Dihydropyridine**	14	15.4
**Digitalis**	11	12.1
**Nitrates**	9	9.9
**Ivabradine**	7	8.7
**Antiplatelet**	56	61.5
**Anticoagulant**	55	60.4

ACEI: Angiotensin-converting enzyme, ARABs: angiotensin II receptor blockers.

**Table 3:** Mean number of hospitalizations for heart failure before and after inclusion in the PRADO-IC program (at 30 days, 6 months, and one year).

Length	Hospitalizations for Heart Failure			Other causes of Hospitalizations		
	Before Inclusion	After Inclusion	P	Before Inclusion	After Inclusion	P
30 days	0.18 (0.42)	0.15 (0.36)	0.56	0.16 (0.37)	0.02 (0.15)	<0.001
6 months	0.98 (1.04)	0.53 (0.81)	<0,001	0.52 (0.72)	0.28 (0.59)	0.006
1 year	1.04 (1.14)	1.04 (1.05)	<0.001	0.92 (1.04)	0.48 (0.78)	0.002

**Table 4:** Mean number of readmissions other than heart failure decompensation and length of hospitalization for heart failure before and after inclusion in the PRADO-IC program (at 30 days, 6 months, and one year).

Length	Readmissions other than HF Decompensation		
	Before Inclusion	After Inclusion	p
30 days	0.02 ± 0.15	0.16 ± (−0.37)	<0.001
6 months	0.52 ± 0.72	0.28 ± 0.59	0.006
1 year	0.92 ± 1.04	0.48 ± 0.78	0.002
	**Length of hospitalization(days) for HF**		
	**Before Inclusion**	**After Inclusion**	**p**
30 days	11.67 ± 4.34	9.77 ± 3.92	0.31
6 months	18.02 ± 7.78	14.28 ± 11.57	0.006
1 year	22,07 ± 10.33	16.39 ± 15.94	<0.001
